# Impact of Fibrin Gel Architecture on Hepatocyte Growth Factor Release and Its Role in Modulating Cell Behavior for Tissue Regeneration

**DOI:** 10.3390/gels10060402

**Published:** 2024-06-16

**Authors:** Svenja Wein, Shannon Anna Jung, Miriam Aischa Al Enezy-Ulbrich, Luca Reicher, Stephan Rütten, Mark Kühnel, Danny Jonigk, Wilhelm Jahnen-Dechent, Andrij Pich, Sabine Neuss

**Affiliations:** 1 BioInterface Group, Helmholtz Institute for Biomedical Engineering, RWTH Aachen University, Pauwelsstrasse 20, 52074 Aachen, Germany; luca.reicher@rwth-aachen.de (L.R.); wjahnen@ukaachen.de (W.J.-D.); sneuss-stein@ukaachen.de (S.N.); 2 Institute of Pathology, RWTH Aachen University, Pauwelsstrasse 30, 52074 Aachen, Germany; mkuehnel@ukaachen.de (M.K.); djonigk@ukaachen.de (D.J.); 3 Functional and Interactive Polymers, Institute of Technical and Macromolecular Chemistry, RWTH Aachen University, Worringerweg 1, 52074 Aachen, Germany; sjung@dwi.rwth-aachen.de (S.A.J.); alenezy@dwi.rwth-aachen.de (M.A.A.E.-U.); pich@dwi.rwth-aachen.de (A.P.); 4 DWI–Leibniz Institute for Interactive Materials, RWTH Aachen University, Forckenbeckstraße 50, 52074 Aachen, Germany; 5 Electron Microscopic Facility, University Clinics, RWTH Aachen University, Pauwelsstrasse 30, 52074 Aachen, Germany; sruetten@ukaachen.de

**Keywords:** tissue engineering, regenerative medicine, Hepatocyte Growth Factor (HGF), fibrin gel scaffolds, controlled release

## Abstract

A novel scaffold design has been created to enhance tissue engineering and regenerative medicine by optimizing the controlled, prolonged release of Hepatocyte Growth Factor (HGF), a powerful chemoattractant for endogenous mesenchymal stem cells. We present a new stacked scaffold that is made up of three different fibrin gel layers, each of which has HGF integrated into the matrix. The design attempts to preserve HGF’s regenerative properties for long periods of time, which is necessary for complex tissue regeneration. These multi-layered fibrin gels have been mechanically evaluated using rheometry, and their degradation behavior has been studied using D-Dimer ELISA. Understanding the kinetics of HGF release from this novel scaffold configuration is essential for understanding HGF’s long-term sustained bioactivity. A range of cell-based tests were carried out to verify the functionality of HGF following extended incorporation. These tests included 2-photon microscopy using phalloidin staining to examine cellular morphology, SEM analysis for scaffold–cell interactions, and scratch and scatter assays to assess migration and motility. The analyses show that the novel stacking scaffold promotes vital cellular processes for tissue regeneration in addition to supporting HGF’s bioactivity. This scaffold design was developed for in situ tissue engineering. Using the body as a bioreactor, the scaffold should recruit mesenchymal stem cells from their niche, thus combining the regenerative abilities of HGF and MSCs to promote tissue remodeling and wound repair.

## 1. Introduction

Tissue engineering and regenerative medicine are groundbreaking fields in modern healthcare, dedicated to repairing and reconstructing damaged tissues using advanced biomaterials and cellular techniques [1]. Central to these methodologies are biocompatible scaffolds, such as fibrin gels, which are derived from natural blood coagulation processes and are designed to emulate the properties of the extracellular matrix. These gels are highly regarded for their exceptional biocompatibility, biodegradability, and seamless integration with host tissues, making them ideal candidates for regenerative applications.

A key factor contributing to the efficacy of these novel scaffolds is HGF, recognized for its potent regenerative abilities, including enhanced cell proliferation, migration, and differentiation [2,3]. The release dynamics of HGF from the scaffolds, influenced significantly by the fibrin gel concentration, are crucial for optimizing the scaffold’s design to maximize tissue repair and regeneration [4]. Nevertheless, the intricate relationship between fibrin gel concentration, HGF release kinetics, and the subsequent cellular responses—such as migration, viability, and overall tissue regeneration—remains insufficiently understood [5].

This study explores the relationship between fibrinogen concentration and the release dynamics of HGF from fibrin gels. By examining various fibrinogen densities, the research evaluates their impact on the kinetics of HGF release and the subsequent effects on primary cells and induced mesenchymal stem cells. These investigations are crucial for refining the design and enhancing the functionality of fibrin-based scaffolds, aiming to optimize their use in tissue engineering and personalized medicine. Additionally, by providing detailed insights into scaffold behavior and its interaction with different cell types, the study contributes to bridging the existing gap between basic research and practical clinical applications, facilitating the development of more effective therapeutic strategies. The study introduces a novel approach that goes beyond the current state of the art by employing a stack of three different fibrinogen concentrations with embedded HGF. Unlike previous methods that rely on single layers and result in a rapid burst release of growth factors, our multi-layered design enables a controlled and sustained release of HGF. This innovative strategy not only enhances scaffold stability but also ensures a prolonged therapeutic effect, creating an optimized healing environment that significantly improves tissue regeneration and overall wound healing outcomes.

**Figure 1:**
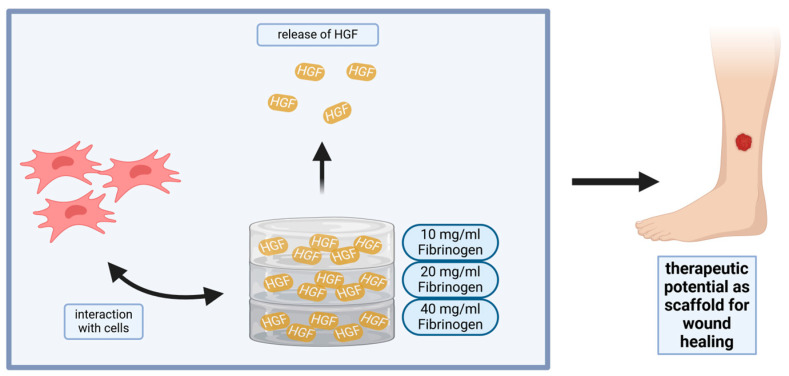
**Engineered release of HGF from fibrin hydrogel scaffolds for enhanced tissue regeneration.** Schematic representation of a study where HGF is released from fibrin hydrogels of different concentrations, highlighting its application in wound healing through the enhancement of cell interaction and regeneration.

To address the existing gap in science concerning the release kinetics of growth factors from fibrin gels, our study explores varying fibrinogen concentrations (10, 20, and 40 mg/mL) with different HGF release properties and introduces an innovative stacked scaffold approach [6]. By layering different fibrinogen concentrations within a single scaffold, we aim to engineer a controlled, long-term release profile for HGF. Figure 1 illustrates the experimental design to prolong the regenerative effects of HGF, in particular the recruitment of endogenous mesenchymal stem cells (MSC), which is vital for complex tissue regeneration scenarios that require sustained intervention.

Hydrogels are indispensable in this context due to their high water content and adaptable biochemical and biophysical properties [7], which distinguish themselves as excellent media for encapsulating and releasing bioactive molecules like HGF. Recent technological advancements have been directed towards adjusting hydrogel properties—such as crosslinking density and polymer composition—to fine-tune the release kinetics of HGF. Precise control over these properties is essential for preserving HGF’s stability and bioactivity, thereby maximizing its therapeutic potential for promoting angiogenesis and extensive tissue growth, which are crucial for the repair of significant tissue damage [8,9].

Additionally, the integration of nanoparticle technology within hydrogels [10,11] serves to protect HGF from premature degradation and facilitates stimulus-responsive release mechanisms. These technological enhancements have demonstrated considerable promise in clinical settings, particularly in accelerating wound healing by promoting re-epithelialization, fibroblast migration, and collagen deposition—processes that are essential for the treatment of chronic wounds where natural healing mechanisms are impaired [12].

A pivotal aspect of our research is its focus on personalized medicine by use of mesenchymal stem cells (MSCs). Our in situ tissue engineering approach heralds a transformative technique within regenerative medicine, circumventing conventional methods of cell isolation and expansion. Harnessing the body’s innate capabilities as a bioreactor, this methodology orchestrates the recruitment and activation of endogenous stem cells, thereby reducing reliance on external cell sources. This pioneering strategy not only simplifies therapeutic procedures but also offers potential for tailoring tissue regeneration to individual patient needs. By harvesting MSCs directly from patients and pre-seeding them on HGF-enhanced scaffolds, cell proliferation could be significantly boosted. This tailored approach not only meets individual patient needs but also facilitates the reimplantation of these bioenhanced scaffolds back into patients, thereby promoting more effective and personalized healing processes with reduced or even without any tissue rejection reaction [13].

Expanding on this concept of personalization, our study utilizes induced MSCs (iMSC) to examine how they benefit from the release of HGF from our stacked hydrogel scaffolds [8]. Utilizing iMSCs, which can be derived from the patient’s own cells, ensures high compatibility and effectiveness for individualized treatments. This strategy not only demonstrates the therapeutic versatility of our scaffolds but also highlights significant enhancements in cellular activity and proliferation induced by HGF, thus expanding the scope of potential therapeutic applications.

The overarching aim of our work is to enhance cell proliferation through the strategic encapsulation of HGF within scaffolds for a variety of applications [2]. These include faster wound healing, for example, for diabetic patients [14]; improved surgical recovery through expedited wound closure; and the advancement of tissue engineering projects such as the construction of organoid scaffolds pre-seeded with cells. These applications greatly benefit from the controlled release of growth factors, which considerably accelerates the cell pre-seeding process and facilitates more rapid tissue regeneration.

By integrating these innovative strategies, our study significantly advances scaffold-based tissue engineering and paves new pathways for developing personalized regenerative therapies. This marks a substantial step forward in the field, pushing the boundaries of what is possible in tissue restoration and regeneration.

### Impact Statement

Our study significantly advances tissue engineering and regenerative medicine by optimizing the release dynamics of HGF from fibrin gel scaffolds. By introducing a stacked scaffold design with varied fibrinogen concentrations and incorporating growth factors, we enhance the timely controlled delivery of bioactive molecules, crucial for tissue repair and regeneration. This approach not only improves scaffold functionality but also aligns with personalized medicine principles by using patient-derived iMSC ensuring high treatment compatibility and effectiveness. These innovations provide foundational insights for clinical applications, particularly in accelerating and especially controlled healing processes and enhancing tissue reconstruction. Our research offers a pivotal step towards the development of next-generation, patient-specific regenerative therapies, marking a transformative contribution to both the scientific community and clinical practice.

## 2. Results and Discussion

### 2.1. Rheological and Structural Properties of Applied Fibrin Gels: Linking Gel Characteristics to Cellular Behavior

In the pursuit of optimizing fibrin gels for biomedical applications, the interplay between their rheological properties and cellular responses is of main importance [15]. This section focuses on an integrated analysis where rheological measurements and structural characteristics of fibrin gels are investigated. The rheological characteristics of fibrin gels at varying concentrations are essential for controlling the diffusion-driven release of HGF. These properties dictate the gel’s porosity and structural density, thereby influencing the kinetics of HGF release, which is critical for optimizing tissue regeneration strategies. Utilizing state-of-the-art rheology techniques and scanning electron microscopy (SEM), an attempt is made to establish a correlation between the physical properties of fibrin gels, such as viscoelasticity and structural integrity, and their effectiveness in supporting cellular functions and behavior [16]. Through a detailed examination of these parameters, contributions are aimed towards the development of fibrin-based materials that are both structurally sound and biologically beneficial.

#### 2.1.1. Rheology

**Figure 2:**
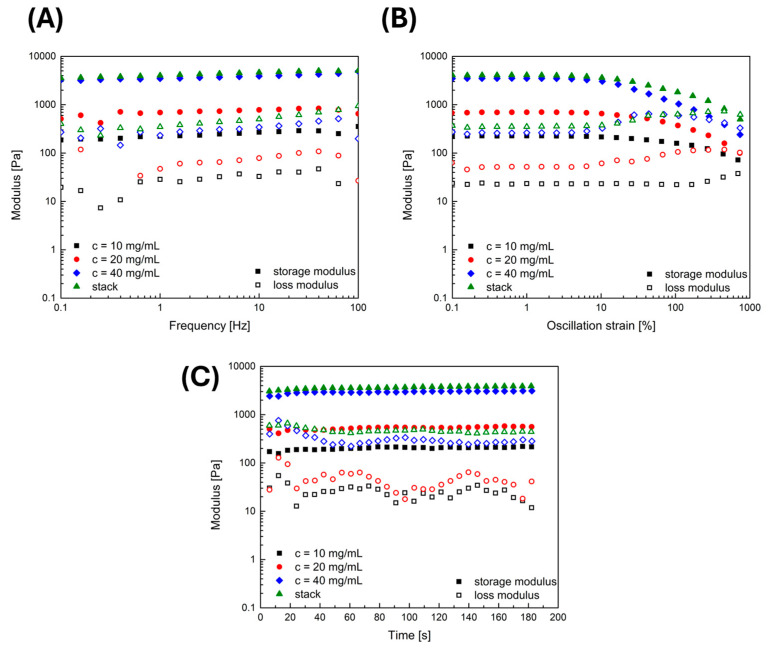
**Rheological analysis of fibrin gel:** (**A**) storage modulus frequency sweep: G′ and G″ of fibrin hydrogels across frequencies (0.1–100 Hz); (**B**) storage modulus vs. oscillation strain: G′ and G″ of fibrin hydrogels at 10, 20, and 40 mg/mL fibrinogen concentrations; (**C**) time-dependent stability: G′ and G″ of fibrin hydrogels over 200 s.

Figure 2 illustrates measurements of rheological properties of fibrin gels with different fibrinogen concentrations, including the novel stacked configuration. Figure 2A, presenting the hydrogels’ moduli as a function of frequency, demonstrates that the moduli are independent on the applied frequency, which is a typical behavior for viscoelastic hydrogels. The storage modulus is highest for the stacked gels and decreases with a decreasing fibrinogen concentration. The decrease in the storage modulus can be explained by less denser gels due to a lower fibrinogen amount in the hydrogel. The results show the storage modulus as a function of frequency, demonstrating that the modulus increases with fibrinogen concentration. The 10 mg/mL fibrinogen gel exhibits the lowest modulus, indicating it has the most flexible and least solid-like properties. In contrast, the 20 and 40 mg/mL fibrinogen gels show higher moduli, implying they possess greater elasticity and are more solid-like, with the 40 mg/mL concentration displaying the highest modulus, reflecting the greatest stability among the individual gels. The stack, a layered combination of all three fibrinogen concentrations, exhibits a modulus similar to the 40 mg/mL fibrin gel, the most rigid of the series. This observation suggests that the stacked gel structure retains the mechanical robustness provided by the highest concentration layer [17,18].

In Figure 2B the strain-dependent measurement indicates strain-softening behavior, which is typical for fibrin-based hydrogels outside the body [7]. This can also be observed for the stacked hydrogels. After the oscillation strain-dependent measurement, a time-dependent measurement was conducted. The time-dependent measurement in Figure 2C shows that the previously performed amplitude sweep does not affect the mechanical properties of the prepared fibrin-based hydrogels. The moduli remain almost constant at 0.1% strain and 1.0 Hz frequency within the whole time-dependent measurement [19].

In tissue engineering, the significance of these findings is manifold. The increasing rigidity with higher fibrinogen concentrations suggests that denser gels could be advantageous for applications requiring mechanical strength to support cellular activities [20]. The stability of the storage modulus over time for each gel concentration and the stacked configuration indicates that these scaffolds can provide a consistent environment, which is vital for supporting the extended release of HGF and for maintaining its regenerative effects during tissue repair processes. The stacked scaffold could offer a stratified structure with varying mechanical properties, potentially guiding cellular behavior and supporting various stages of tissue regeneration.

Collectively, the rheological properties align well with the desired attributes for regenerative therapies, wherein the scaffold must offer both stability and support for cell growth and extracellular matrix deposition [21]. The results from these rheological assessments complement the conducted cell-based assays and morphological evaluations, collectively underscoring the potential of this innovative scaffold design.

#### 2.1.2. SEM Analysis

**Figure 3:**
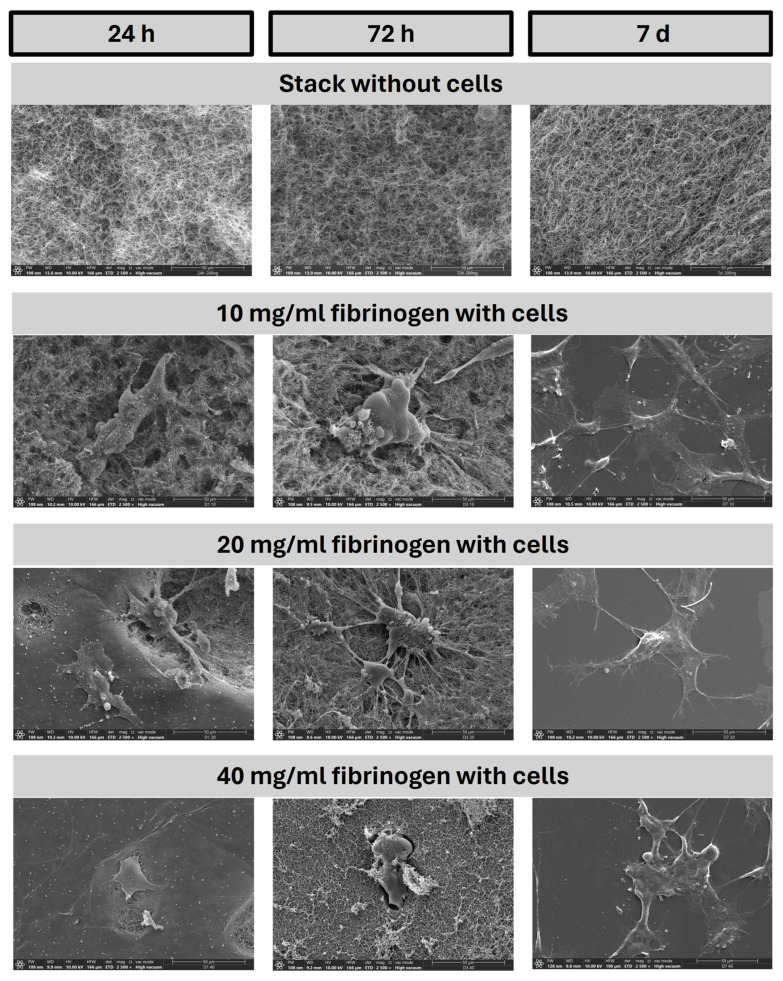
**Cellular integration and scaffold architecture over time.** The image shows a series of scanning electron microscopy images comparing the structural integrity and cellular activity within fibrin hydrogel scaffolds over time intervals of 24 h, 72 h, and 7 days, with varying fibrinogen concentrations. It illustrates the scaffolds both without cells to demonstrate scaffold architecture and with cells to show cellular adhesion and proliferation. Magnification 2500×.

Figure 3 comprises a series of SEM representations images representing the degradation over a one-week period of fibrin gels both alone and in conjunction with cells. The stack of three fibrinogen conditions without cells demonstrates a stable, porous structure that exhibits little alteration over the time course observed. The cell-free scaffold showed no significant signs of biodegradation after 7 days of incubation. This can be attributed to the intrinsic stability of the scaffold material, which is designed to maintain its structural integrity in the absence of cellular activity. Environmental conditions such as temperature, pH, and moisture levels during the experiment might not have been optimal for promoting hydrolysis. Additionally, the 7-day period may not be sufficient to observe substantial hydrolysis, indicating that a longer observation period might be necessary to detect noticeable degradation. When cells are incorporated into the fibrin matrix at the same concentration, a distinct shift in the structural integrity of the gel is observed. Cells are seen beginning to adhere and interact with the fibrin network [22]. After 72 h, the cell spreading throughout the matrix becomes more evident, indicating active engagement with the gel. At the seven-day point, the fibrin network displays signs of degradation, presumably due to cellular activity. This degradation process is crucial, as it not only indicates the breakdown of the scaffold but also suggests the potential release of bioactive molecules, such as HGF, which may be embedded within the gel. The controlled nature of this degradation implies a mechanism through which HGF can be gradually released, resulting in cell recruitment and tissue remodeling [23]. With an increase in fibrinogen concentration to 20 and 40 mg/mL where cells are present, a denser fibrin network is initially observed. Despite this increased density, cell adhesion and the subsequent remodeling of the scaffold are evident. The fibrous structure of the scaffold at 24 h for the 20 mg/mL and 40 mg/mL fibrinogen samples is present, but less distinctly visible compared to other conditions. This is likely due to the denser matrix formed at higher fibrinogen concentrations, which can obscure individual fibers. Additionally, the interaction of cells with the scaffold and the resolution settings of the SEM images may influence the visibility of finer fibrous details. The initial cellular attachment and interaction are clearly demonstrated in these images. As time progresses, particularly in the densest scaffold of 40 mg/mL, cellular activity is maintained, suggesting that higher scaffold densities do not impede cellular functions. By day 7, a noticeable diminishment in the fibrin network is apparent across all cell-incorporated concentrations, indicating ongoing degradation of the gel. This activity is indicative of a process that may facilitate the sustained release of HGF, a pivotal element in supporting regenerative processes within tissues. In essence, these SEM images capture the dynamic interactions between the fibrin scaffold and cells, illustrating the compatibility of the scaffold with cellular functions and its ability to degrade in a manner that likely promotes the gradual release of therapeutic agents, vital for the field of tissue engineering and regenerative medicine [24].

To complement the qualitative SEM observations, our working group has previously conducted a detailed quantitative analysis of cell adhesion and proliferation on fibrin hydrogels. This analysis included precise measurements of cell adhesion rates and proliferation indices, providing robust quantitative data that support the observed cell behaviors. These characterizations affirm the bioactivity and suitability of the fibrin hydrogels for promoting cellular activities critical for tissue regeneration [25].

### 2.2. Decoding Fibrin Gel Degradation Dynamics and Hepatocyte Growth Factor Release

**Figure 4:**
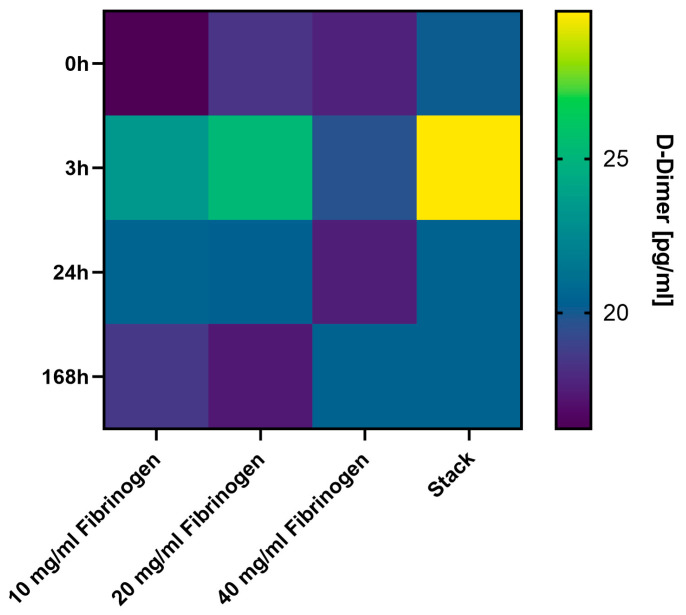
**D-Dimer release profile from fibrin hydrogel scaffolds.** The image displays a heatmap illustrating the concentration of D-Dimers, fibrin degradation products, released from fibrin hydrogel scaffolds at different time points (0 h, 3 h, 24 h, 168 h) and across varying fibrinogen concentrations (10, 20, 40 mg/mL) and a stacked combination. The color gradient indicates the quantity of D-Dimers present, serving as an indicator of scaffold degradation and HGF release kinetics over time.

**Figure 5:**
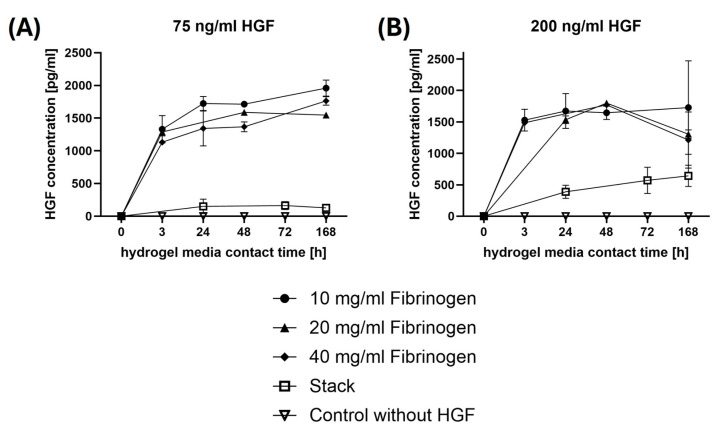
**Release patterns of HGF from fibrin hydrogels over time.** The graphs (**A**,**B**) illustrate the concentration of HGF released from fibrin hydrogels over time, measured at initial loadings of 75 ng/mL and 200 ng/mL HGF, respectively. They compare the release profiles across different fibrinogen concentrations and a stacked scaffold, with a control showing no HGF release.

The degradation dynamics of various fibrin gel compositions and the corresponding release kinetics of HGF were examined. Figure 4 illustrates the degradation behavior analyzed by D-dimer ELISA, which quantifies the breakdown products of fibrin as the gels degrade over time [26]. Figure 5 illustrates the corresponding release of HGF from these gels, providing insight into the time-dependent HGF delivery capabilities of the fibrin matrices. This dual analysis aims to elucidate how the structural and compositional nuances of fibrin gels influence both their degradation pathways and their function as carriers for bioactive molecules like HGF.

#### 2.2.1. D-Dimer ELISA

The heatmap from the D-Dimer ELISA assay shown in Figure 4 provides a detailed picture of the degradation behavior of fibrin gels under four different conditions across a time of one week. At 0 h, the assay reveals a uniform D-Dimer level across all samples, as is expected in the absence of any degradation. At the 3 h mark, D-Dimer levels peak, particularly in the stacked gel configuration, suggesting rapid initial degradation of the fibrin matrix. The higher biodegradation observed in the stack group at the 3 h mark compared to other groups can be attributed to the initial burst release phenomenon. This phenomenon is common in scaffold materials, where the surface-bound components degrade rapidly upon initial exposure to the medium, leading to a transient spike in degradation products. This early surge in D-Dimer could be attributed to an immediate response to the environment or the activation of degradation processes within the gel. This peak may also indicate the beginning of the release of HGF if it is incorporated into the matrix, an event that could be beneficial for early-stage tissue repair processes where a burst release of HGF might be desirable to kick-start cellular activities. By 24 h, the levels of D-Dimers begin to vary more distinctly among the different fibrinogen concentrations, with some showing a higher degree of degradation than others. This variation could be due to the inherent properties of the fibrin gels at different concentrations affecting their stability and susceptibility to enzymatic breakdown [26]. The observed similar degradation levels across different fibrinogen concentrations at the same time points suggest that the initial concentration of fibrinogen may not significantly influence the rate of degradation within the first 168 h. This could indicate that other factors, such as environmental conditions or the intrinsic properties of the scaffold, play a more dominant role in the early stages of biodegradation. By the end of the observation period at 168 h (7 days), the continued degradation is evidenced by the persistent presence of D-Dimers, although the levels seem to stabilize or slightly decline from the initial peak at 3 h. This indicates that the most intense phase of degradation occurs quite early, followed by a more gradual breakdown of the fibrin matrix over time. The sustained presence of D-Dimers at this point is consistent with a controlled release mechanism for HGF, ensuring its availability over the entire duration of the healing process. The stacked gel configuration presents an intriguing pattern of degradation. The initial rapid increase in D-Dimer levels followed by a stabilization suggests that the layered structure may influence the rate and extent of gel breakdown. This could imply that different layers degrade at different rates, possibly providing a staggered release profile for HGF that could be tuned to match the stages of tissue regeneration. In summary, the precise analysis of the heatmap shows that the degradation of fibrin gels and the corresponding release of HGF can be intricately managed by varying fibrinogen concentrations and by using a stacked scaffold design. The early peak in D-Dimer levels at 3 h followed by a stabilization suggests a promising avenue for tailoring the release of regenerative factors in a way that can be synchronized with the body’s natural healing processes.

#### 2.2.2. HGF-Elisa

Figure 5 illustrates the release kinetics of HGF from fibrin gels of different fibrinogen concentrations and a stacked gel configuration over time, with two initial HGF concentrations: 75 ng/mL and 200 ng/mL. Figure 5A shows that the release of HGF from gels with an initial concentration of 75 ng/mL follows a trend where the amount of HGF detected in the medium generally increases over time. This trend is most pronounced in the 10 and 20 mg/mL fibrinogen gels, which demonstrate a more sustained release up to 168 h (7 days). The 40 mg/mL fibrinogen gel also exhibits a release pattern, but it levels off more quickly, indicating a possible early saturation or reduced release rate. The stacked gel shows a moderate and sustained release, but not as high as the lower concentrated gels. The stack approach exhibits a slower HGF release compared to a single layer, as the increased thickness of the gel results in more gradual degradation, thus facilitating a more controlled and steady release of HGF without a burst effect. The control without HGF, as expected, shows no release.

Figure 5B represents a higher starting concentration of 200 ng/mL HGF, resulting in a different release profile. Initial release within the first 24 h is most pronounced in the 10 mg/mL fibrinogen gel. Release peaks at 48 h, particularly in the 20 and 40 mg/mL fibrinogen gels. The release then decreases or plateaus towards 168 h. The stacked gel in this instance exemplifies our precise design goals, showcasing a flawless, consistent release profile devoid of any peaks. This achievement solidifies its suitability for therapeutic applications by effectively circumventing the conventional burst release pattern, ensuring a robust and uninterrupted delivery of bioactive agents. The control shows no release, confirming the specificity of the HGF ELISA. When considering the normal amount of HGF present in the human body or in wound-healing applications, the physiological concentration of HGF can vary. In normal serum, HGF levels can be relatively low, typically under 0.5 ng/mL [27,28,29], but may increase significantly in tissue repair and wound-healing scenarios up to 140 ng/mL [30,31]. In therapeutic contexts, the concentrations of HGF could be elevated to promote healing. Therefore, the release amounts detected from the fibrin gels, especially in the higher initial concentration setup, are well above normal serum levels but may align with the elevated levels needed in therapeutic applications to ensure efficacy [32,33]. The initial burst of HGF release from the gels may result in kick-starting the healing process by providing a high concentration of HGF at the beginning, which can be beneficial for attracting cells to the wound site. The subsequent sustained release, particularly evident in the stacked gel configuration, would be valuable in maintaining a conducive environment for ongoing tissue repair and regeneration. This controlled release profile, especially with the stacked gel, may allow for prolonged bioactivity of HGF, aligning with the extended time frames required for complex tissue regeneration. The data suggest that by manipulating the fibrinogen concentration and the scaffold structure, the release of HGF can be tailored to match the therapeutic needs of tissue engineering and regenerative medicine. 

The initial burst release of HGF observed in certain configurations (Figure 5) has significant therapeutic implications for tissue engineering and regenerative medicine. This initial surge can start the healing process by attracting cells to the wound site. However, the rapid depletion of HGF could reduce therapeutic efficacy over time.

The stacked gel configuration offers a solution with its slower, controlled release profile, ensuring a continuous supply of HGF necessary for prolonged tissue repair. Adjusting the fibrinogen concentration and scaffold structure allows tailoring of HGF release to meet specific therapeutic needs, enhancing clinical applications.

In conclusion, the controlled initial burst and sustained release of HGF from fibrin gels present a promising strategy for effective tissue repair and regeneration, aligning well with the dynamic requirements of the healing process.

### 2.3. Evaluating the Bioactivity of Hepatocyte Growth Factor Released from Fibrin Gels

Next the research delves into the functional assessment of HGF through two distinct cell-based assays: the scratch and scatter assays [34]. The foundational approach for both assays involved conditioning cell media by allowing it to interface with fibrin gels under a range of conditions for a 24 h period. This step was crucial to ensure that the medium acquired the HGF released from the gels. Following this conditioning phase, the medium was then introduced to cell cultures to evaluate the influence of HGF on cellular behaviors indicative of bioactivity.

It is important to note that in this investigative stage, the stacked scaffold configuration was not subjected to the indirect assays. The assumption behind this decision stemmed from the understanding that within the short span of the 24 h conditioning period, the deeper layers of the stack would unlikely undergo significant degradation. Consequently, the release of HGF was expected to be primarily from the topmost layer of 10 mg/mL fibrinogen. Additionally, initial findings from HGF Elisa (Section 2.2) assays indicated that the release of HGF from the stacked scaffolds was not as pronounced, reinforcing the decision to focus on the single-layer configurations for the current bioactivity evaluations. Future studies are anticipated to extend the analysis to the stacked scaffolds, pending a comprehensive profiling of the release kinetics of HGF from each individual layer within the scaffold.

#### 2.3.1. Scratch Assay

**Figure 6:**
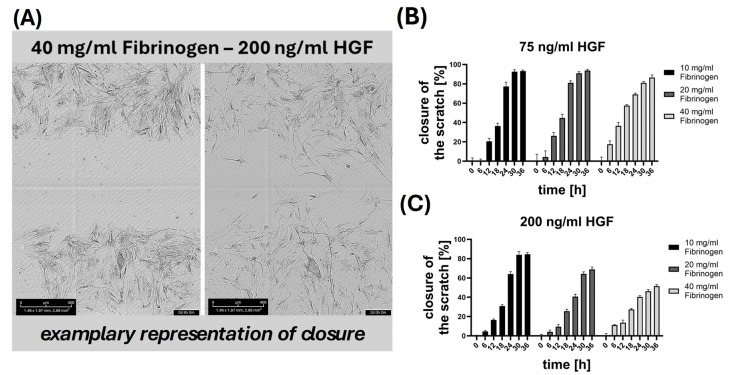
**Scratch assay using MSC, and conditioned media released from HGF-containing fibrin hydrogels.** (**A**) depicts a microscopic image showing MSC migration and scratch closure in fibrin hydrogels containing 40 mg/mL fibrinogen and 200 ng/mL HGF at 6 h (left) and 2 days (right) post scratching. Scale bar 400 µm. (**B**,**C**) present bar graphs quantifying the percentage of scratch closure over time in hydrogels with varying fibrinogen concentrations, at two different HGF concentrations (75 ng/mL and 200 ng/mL).

To assess the bioactivity of HGF released from fibrin gels, a scratch assay was employed [35]. The conditioned medium was applied to scratched MSC cultures to study cell migration, a critical indicator of HGF/scatter factor biological activity. Figure 6A displays a typical view of a scratch assay, showing the initial scratch in the MSC monolayer (left) and scratch closure after 24 h in the presence of conditioned media from the 40 mg/mL fibrinogen gel with 200 ng/mL HGF. MSC largely bridged the scratch area over 24 h suggesting that the HGF contained in the conditioned media had facilitated cell migration. 

Figure 6B,C show the percentage of scratch closure in the presence of conditioned media from gels initially containing HGF concentrations of 75 ng/mL and 200 ng/mL, respectively. For the 75 ng/mL HGF condition shown in Figure 6B, there is a progressive increase in closure percentage, indicating that the HGF released into the media is stimulating cell migration. Interestingly, the conditioned media released from gels containing 10 and 20 mg/mL fibrinogen mediated better scratch closure than conditioned media released from gels containing 40 mg/mL fibrinogen, suggesting higher HGF release from less dense fibrinogen gels. Figure 6C illustrates the 200 ng/mL HGF condition, showing that higher HGF concentrations may initially induce a more robust migration; this effect, however, appears to diminish over time, particularly in higher fibrinogen concentrations. This could be due to a denser fibrin network at higher concentrations potentially restricting the release or diffusion of HGF into the media. It could be observed that cell migration appears more pronounced in the presence of 75 ng/mL HGF (Figure 6B) compared to 200 ng/mL HGF (Figure 6C). It can be hypothesized that this finding may be attributed to the optimal concentration of HGF required for maximal cell migration. Specifically, a concentration of 75 ng/mL HGF might create a more conducive environment for cell migration, whereas higher concentrations, such as 200 ng/mL, could potentially lead to saturation effects or trigger inhibitory mechanisms.

The inclusion of a control group without HGF would provide a baseline for cell migration; this aspect has been comprehensively addressed in a previous publication from our working group [4]. In that study, the baseline cell migration in the absence of HGF was thoroughly characterized, offering a well-documented framework for comparison. By leveraging the established baseline from our previous work, we focused our current study on examining the comparative effects of varying concentrations of fibrinogen and HGF. This approach allows for a more targeted investigation into the impact of HGF on cell migration.

The scratch assay results support the conclusion that HGF is not only released from the fibrin gels and is also biologically active in stimulating MSC migration. Moreover, it highlights the importance of fibrinogen concentration in modulating HGF release, suggesting that lower concentrations may be more effective for sustained HGF activity and thus better suited for applications where prolonged growth factor activity is needed for tissue regeneration.

The presented results show that HGF released from fibrin gels stimulated MSC migration in a dose-dependent manner. Lower fibrinogen concentrations (10 and 20 mg/mL) facilitated better HGF release and sustained cell migration compared to denser gels (40 mg/mL), which restricted HGF diffusion over time. Overall, the results demonstrate a clear dose-dependent relationship between fibrinogen concentration and cell migration, with higher fibrinogen levels leading to slower migration rates, likely due to decreased HGF availability. One of the biggest challenges of this approach lies in the interaction and decision making regarding which fibrin gel concentration and HGF concentration to choose. The optimal combination depends heavily on the desired cell migration profile. If rapid, short-term availability of HGF is required, lower fibrinogen concentrations may be preferable, as they allow for quicker HGF release and faster cell migration. Conversely, if a long-term release of HGF with a steady, slow start is desired, higher fibrinogen concentrations may be more suitable, as they provide a slower release of HGF and consequently a more gradual cell migration response. The dose-dependent effect on the cells is critically influenced by these choices, determining the overall effectiveness of the cell migration strategy.

#### 2.3.2. Scatter Assay

**Figure 7:**
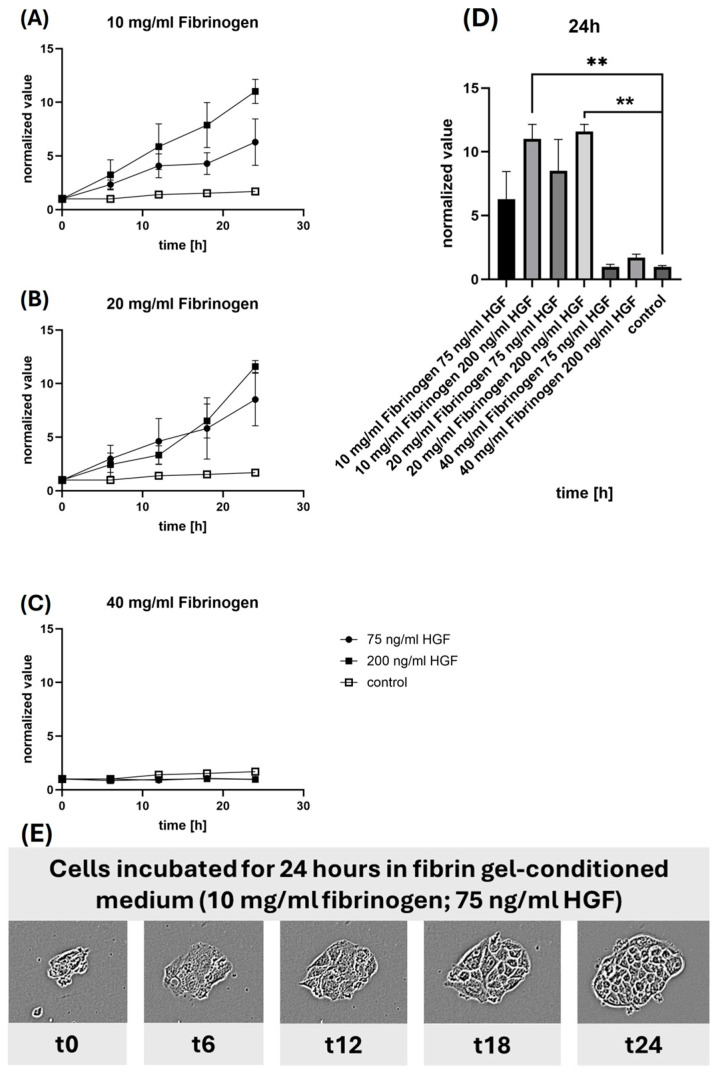
**Cell scatter fibrinogen concentration gradients.** The scatter plots (**A**–**C**) show the normalized value of cell scatter at varying fibrinogen concentrations with different HGF doses over time, and (**D**) compares these rates at 24 h, highlighting the statistically significant differences. The sequence (**E**) displays exemplary the morphological changes of cells incubated in a fibrin gel-conditioned medium over 24 h, demonstrating the effects of fibrinogen and HGF on cell behavior. (**: statistically high significant difference).

An established cell scatter assay was employed to assess cell motility and dispersion, simulating salient parts of wound healing and tissue regeneration [36]. Figure 7 shows the results of the scatter assay using MDCK cells that were preconditioned with medium that equilibrated with HGF-containing fibrin gels of varying fibrinogen concentration.

Figure 7A shows that conditioned medium from HGF containing fibrin gels made from 10 mg/mL fibrinogen increased cell dispersion dose-dependently, with 200 ng/mL HGF doubling the scatter over 75 ng/mL HGF. As expected, control MDCK cultures without added HGF exhibited little cell dispersion, underscoring the stimulatory role of HGF.

Figure 7B demonstrates a similar pattern for the 20 mg/mL fibrinogen concentration, with cell dispersion again being higher in the medium conditioned with 200 ng/mL HGF. The effect is less pronounced than with the 10 mg/mL fibrinogen, indicating that the denser fibrin network significantly affects the release and activity of HGF.

Figure 7C illustrates results obtained with conditioned media obtained from fibrin gels prepared with 40 mg/mL fibrinogen. Little if any cell scatter was observed with both HGF conditions, suggesting strongly restricted release of HGF due to the high density of the fibrin gel or an inhibition of cell motility due to the physical properties of the gel.

Figure 7D compares data obtained from all fibrinogen concentrations. Here, the impact of different concentrations of HGF is evident, with the highest dispersion seen in cells treated with media conditioned by 10 mg/mL fibrinogen with 200 ng/mL HGF. The increased dispersion correlates with a higher concentration of HGF, which suggests that the stimulatory effect of HGF is preserved and that the release from the gel is functional. It is also noticeable that the control without HGF shows very low dispersion, which further confirms the role of HGF in stimulating cell motility.

Due to the enormous number of data generated by a threefold approach, only an exemplary data series of the Cell Scatter is shown in this context. The series of images shown in Figure 7E captures exemplary the morphological changes in MDCK cells over 24 h when incubated with medium conditioned by 10 mg/mL fibrinogen gel containing 75 ng/mL HGF. The progressive increase in the scattered area indicates active cell motility, reinforcing the results from the graphs that HGF promotes cell movement.

The data from these scatter assays support the conclusion that the HGF released from the fibrin gels retains its biological function and is capable of inducing cell motility, an essential factor in wound healing and tissue regeneration [36]. Furthermore, the results highlight the importance of fibrinogen concentration and HGF dosage in designing scaffolds for regenerative medicine, as they can influence the extent of HGF bioactivity and thus the therapeutic efficacy.

To further confirm the functional integrity of the released HGF over time, the results from both the scratch and scatter assays were carefully analyzed. The consistent cell migration observed in the scratch assay, particularly with lower fibrinogen concentrations, underscores the sustained bioactivity of HGF. Similarly, the scatter assay results with MDCK cells, demonstrating increased cell dispersion with higher HGF concentrations from less dense fibrin gels, further validate that the HGF retains its bioactivity post-release. These findings collectively confirm that the HGF released from the fibrin gels remains biologically active, effectively promoting cellular behaviors essential for wound healing and tissue regeneration over the assessed period. The data from these assays indicate that the released HGF is functionally intact, supporting its potential therapeutic applications.

### 2.4. Assessment of Fibrin-HGF Gels as Scaffold Materials: Morphological Insights from Two-Photon Microscopy

In the assessment of fibrin-HGF gels as scaffold materials, morphological insights gained from two-photon microscopy offer an insight into the interaction between iMSC and the scaffold environment [37]. We employed iMSCs given their potential for differentiation into various cell types, thus offering a versatile platform for assessing the efficacy of regenerative therapies in view of their potential application in personalized medicine and tissue engineering.

#### Two-Photon-Microscopy

**Figure 8:**
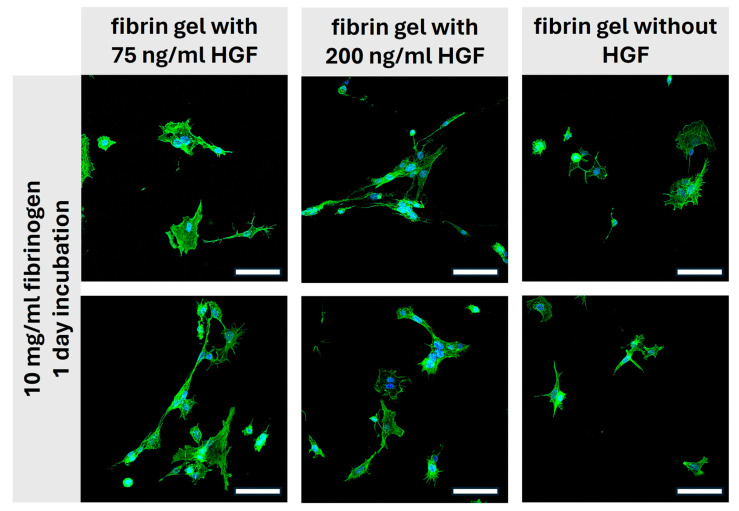
**Cell morphology in fibrin gels: impact of HGF concentration after 24 h.** The images demonstrate fluorescence microscopic views of cells after a 1-day incubation in 10 mg/mL fibrinogen hydrogels with varying concentrations of HGF including gels without HGF. These visualizations highlight the influence of HGF on cell structure and extension, with more pronounced cell spreading and connectivity in the presence of higher HGF concentrations. Scale bar 100 µm. (Staining: Phalloidin-488, green; DAPI: blue).

Figure 8 shows iMSC after one day of incubation in medium conditioned with different concentrations of HGF embedded within 10 mg/mL fibrin gels. The first column of images, showing cells incubated with 75 ng/mL HGF, reveals moderately spread cells with some extension of processes, suggesting that this concentration of HGF supports cellular adherence and initial spreading. However, the morphology does not indicate extensive migration or proliferation at this stage. In the second column with a higher HGF concentration of 200 ng/mL, there is a notable difference. Cells exhibit more pronounced spreading with extensive filopodial and lamellipodial extensions. This enhanced cellular behavior indicates that the increased concentration of HGF significantly fosters not only cell attachment but also promotes cytoskeletal organization, which is crucial for cell migration and tissue integration [38]. In contrast, the third column without HGF shows cells with less spreading and fewer extensions compared to the HGF-incubated cells. These cells display a more rounded morphology, a typical characteristic when cell–matrix interactions are minimal or when growth factors that stimulate spreading and motility are absent. After one day of incubation, the results collectively suggest that HGF has a potent effect on the behavior of iMSCs, enhancing cell spreading and morphological complexity, which are indicative of active cellular engagement and a positive response to the scaffold environment. This qualitative analysis underscores the potential of HGF-incorporated fibrin gels in promoting cellular functions necessary for effective tissue engineering and personalized medicine applications. It highlights the importance of growth factor concentration in influencing iMSC behavior, which could be crucial for the design of tailored regenerative therapies. In general, however, the differences between the samples with and without HGF are not yet significant after one day of incubation, as the fibrin hydrogel provides a good basis for cell growth even without incorporation of HGF.

**Figure 9:**
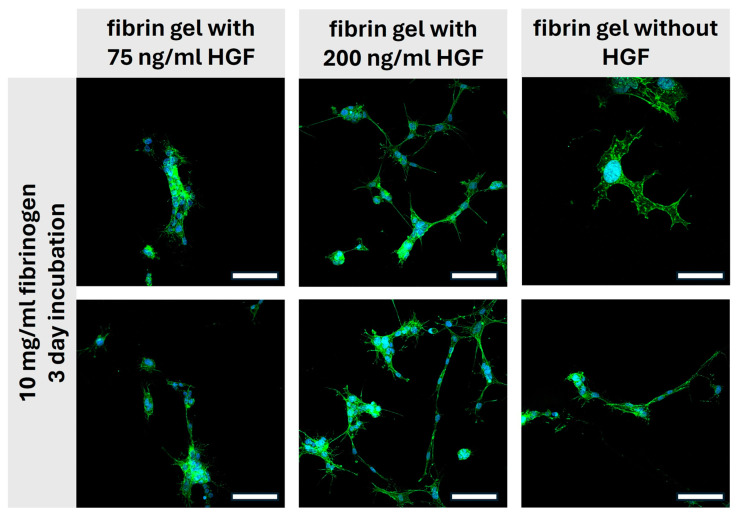
**Cellular dynamics in fibrin gels after 3-day HGF exposure.** The fluorescent images display the morphological progression of cells in 10 mg/mL fibrinogen hydrogels after a 3-day incubation period with two concentrations of HGF (75 ng/mL and 200 ng/mL) and without HGF. These images highlight the differences in cell network formation and interaction within the scaffold environment influenced by the presence and concentration of HGF. Scale bar 100 µm. (Staining: Phalloidin-488, green; DAPI: blue).

In this collection of images from the three-day incubation experiment, we observe the effects of prolonged exposure to different concentrations of HGF on iMSC within fibrin gels (Figure 9).

Starting with the first column, where the iMSCs are incubated with 75 ng/mL HGF, there is an evident progression in cell morphology from day 1. The cells show increased spreading and the development of more complex networks of extensions, which suggests active cell-matrix interactions. The extended processes are indicative of enhanced cellular motility and the establishment of a more interconnected cellular network, which may be critical for tissue formation and integration [39].

The second column, with a higher HGF concentration of 200 ng/mL, shows a more pronounced advancement in cellular behaviors compared to the 1-day incubation. The iMSCs here have established a much denser network of extensions, and their overall morphology appears more differentiated. This suggests that a higher concentration of HGF continues to play a significant role in cellular dynamics over a longer period, promoting robust cytoskeletal organization and potentially influencing differentiation pathways [39].

In contrast, the third column without HGF exhibits the least complex morphologies, with cells remaining relatively rounded and displaying minimal extensions even after three days. This persistence of a less differentiated state underlines the crucial role of HGF to promote signaling pathways that regulate cell behavior. Without it, cells seem to maintain a more quiescent state, which may impede the processes necessary for effective tissue regeneration.

Over three days of incubation, it is apparent that the presence and concentration of HGF in fibrin gels greatly affect the morphological and functional progression of iMSCs. Higher concentrations of HGF lead to a more interconnected and complex cellular network, suggesting an optimized scaffold environment for tissue engineering purposes [39]. This observation is a valuable information for the design of regenerative therapies by emphasizing the significance of growth factor concentration over time to support the desired cellular activities for tissue repair and regeneration.

Comparing day 1 to day 3, there is a significant advancement in cell morphology and behavior, especially when considering the influence of HGF [36,40]. On day 1, cells generally display moderate spreading and initial extension development, particularly evident in the presence of HGF. However, by day 3, this progression is notably more pronounced, with cells showing increased spreading, more complex networks of extensions, and denser morphologies, especially with higher concentrations of HGF. This suggests a continued influence of HGF over time, promoting enhanced cellular motility, cytoskeletal organization, and potentially influencing differentiation pathways for tissue engineering applications. In contrast, the cells incubated without HGF display the least complex morphologies, remaining relatively rounded and displaying minimal extensions even after three days. This emphasizes the critical role HGF plays in stimulating cellular activities necessary for effective tissue regeneration, highlighting the significance of growth factor concentration over time in scaffold design for regenerative therapies.

The observed morphological changes indicate that HGF enhances cell spreading and network formation within the fibrin gels. Higher HGF concentrations promote more extensive and complex cellular networks, suggesting improved cell–cell interactions and migration. These morphological changes imply that scaffolds with higher HGF concentrations may better support tissue regeneration in vivo by facilitating scaffold integration with surrounding tissues, leading to more effective healing and tissue repair processes. Future studies will focus on quantifying these morphological changes through a more robust three-point approach to validate these observations and further elucidate the role of HGF in scaffold-based tissue regeneration.

## 3. Conclusions

In conclusion, we investigated the potential of fibrin gels with different fibrinogen concentrations embedded HGF as scaffold materials for tissue engineering and regenerative medicine. Through a comprehensive series of experiments and analyses, we have gained valuable insights into the mechanical properties, degradation behavior, and bioactivity of these innovative scaffold designs.

In this study it could be observed that fibrin gels with lower concentrations degraded more rapidly. Conversely, a layered stack comprising all three concentrations exhibited a slower, more controlled degradation. Importantly, the released HGF maintained its functionality, effectively enhancing cell proliferation and promoting wound closure.

Rheological assessments revealed the excellent suitability of the fibrin gels for use as scaffold materials, demonstrating controlled release profiles of HGF, a crucial factor for recruiting cells essential for tissue regeneration [41,42]. Moreover, D-Dimer ELISA assays provided insights into the degradation kinetics of the fibrin gels, confirming their ability to undergo controlled degradation over time, facilitating the release of embedded HGF.

Functional testing, including scratch and scatter assays, demonstrated the bioactivity of the released HGF in conjunction with different conditions of the fibrin layers [43], promoting cellular migration, motility, and cytoskeletal organization, all crucial processes for tissue repair and regeneration [39,40]. Furthermore, two-photon microscopy offered qualitative insights into the morphological changes of iMSC within the fibrin gels with embedded HGF, highlighting the influence of HGF concentration on cellular behavior and network formation.

Overall, our findings underscore the potential of fibrin gels with embedded HGF as promising scaffold materials for tissue engineering applications. By optimizing growth factor concentration and scaffold composition, these scaffolds can be tailored to support specific cellular activities necessary for effective tissue regeneration. Moving forward, further investigations into the long-term effects and in vivo efficacy of these scaffold designs will be crucial for advancing personalized regenerative therapies and addressing the complex challenges in tissue engineering.

This study’s in vitro focus on the role of fibrin gel architecture in HGF release provides valuable insights, though it necessitates further in vivo exploration to enhance real-world applicability. While concentrating on a single stem cell type, maintaining consistency, future research should expand the cell models used to validate and generalize our findings. Acknowledging unexplored challenges and limitations could also strengthen the robustness of our conclusions.

Our work is pioneering, opening new pathways in regenerative medicine by exploring how biomaterial structures can influence biological responses—an area that holds potential for significant clinical advancements. The specific use of one stem cell type sets a clear baseline for future studies to build upon, potentially exploring various cellular processes and mechanical properties of gels.

By pushing the boundaries of biomaterials science, our research lays a foundation for future innovations that could transform therapeutic approaches, enhancing both the scope and impact of tissue engineering.

## 4. Materials and Methods

### 4.1. Cells

Mesenchymal stem cells MSC were isolated and cultured following established protocols [43]. In short, cells were isolated from human bone spongiosa, which had to be removed during orthopedic surgery. The cells were selected by adherent cell culture in MSC maintenance medium. Their identity was confirmed by flow cytometry and differentiation potential in osteogenic, chondrogenic and adipogenic differentiation media. Cells were used up to passage number 6.

Madin-Darby canine kidney (MDCK) cells are a model mammalian epithelial cell line used to test HGF/scatter factor bioactivity. In short, the cells were cultured in DMEM medium, and cell scattering was induced with pure HGF or with medium equilibrated with HGF-containing fibrin hydrogels.

Induced MSC were generated from MSC by project partners of Maastricht University.

### 4.2. Fibrin Hydrogels with and without HGF

The preparation involved two main components: buffer solutions and specific experimental setups. For the buffer solutions, a 50 mM solution of CaCl_2_ was prepared by dissolving in distilled water and was subsequently sterilized through filtration. The GBSH_5_ buffer was made in two forms: an incomplete version without D-Glucose and a complete version with 4.5 g/L D-Glucose added. The incomplete GBSH_5_ contained KCl, MgCl_2_, MgSO_4_, NaCl, Na_2_HPO_4_, and HEPES in specified concentrations.

In terms of experimental preparations, the dialysis tubing was sterilized by heating in a solution of 2% NaHCO_3_ and 1 mM EDTA at pH 8, followed by a rinse with sterile water for injections and a second heating in a 1 mM EDTA solution. The tubing was stored in 1 mM EDTA at 4 °C and rinsed before each use. For the fibrinogen suspension with 40 mg/mL 500 mg of fibrinogen powder (62% protein, Sigma Aldrich/Merck, St. Louis, MO, USA) was dissolved in 6.25 mL of sterile water and mixed with an equal volume of the incomplete GBSH_5_ buffer. This mixture was transferred into the sterilized dialysis tubing and stored in the incomplete GBSH_5_ buffer. It was equilibrated overnight at 4 °C, centrifuged, and the supernatant was sterile filtered. For different concentrations, dilutions were made to achieve 10 and 20 mg/mL using the prepared fibrinogen solution and incomplete GBSH_5_ buffer. The prepared aliquots were stored at −80 °C and thawed as needed.

A fibrinogen buffer suspension was prepared according to Table 1. To prepare the gels in 48-well plates, thrombin was transferred to the bottom of the well and fibrinogen buffer suspension was added and mixed quickly. Gels were incubated at 37 °C for 15 min before further usage.

For the experiments involving HGF, recombinant human HGF derived from HEK293 cells was used. Specifically, 25 µg of the HGF was dissolved in GBSH5 buffer to prepare two stock solutions with concentrations of 1000 ng/mL and 2000 ng/mL, respectively. These stock solutions were then utilized for further experimental applications as required.

### 4.3. Mechanical Properties of Fibrin Gels: Rheology

Rheological measurements were performed to assess the mechanical properties of the hydrogels using a TA Instrument Discovery HR-3 hybrid rheometer. This equipment features a 20 mm parallel plate geometry and is equipped with a solvent trap. The previously prepared hydrogels (13 mm diameter) were positioned between the geometry and the Peltier plate. The gap was adjusted to 200 µm and the temperature was set to 37 °C. To prevent evaporation and drying, the solvent trap was filled with water, and the setup was sealed with a lid. First, a time-dependent measurement for 180 s at a frequency of 1.0 Hz and an oscillation strain of 0.1% was conducted to incubate the gel. Then, a frequency-dependent measurement at a fixed strain of 0.1% was performed. The frequency was increased from 0.01 Hz to 100 Hz. This was followed by a strain-dependent measurement at a fixed frequency of 1.0 Hz and an increasing strain from 0.1% to 1000%. Lastly, another time-dependent measurement for 180 s at a fixed strain of 0.1% and a fixed frequency of 1.0 Hz was conducted.

### 4.4. Quantification of D-Dimer and HGF Levels via ELISA

The ELISA assays were performed using the supernatant medium, strictly following the protocols specified by the manufacturers for each kit. The first assay employed the D-Dimer Human ELISA Kit (Invitrogen, Waltham, MA, USA, Catalog # EHDDIMER), which is optimized for accurate detection of D-Dimer levels in human samples. The second assay utilized the Human HGF ELISA Kit (Sigma-Aldrich, RAB0213), designed for cell and tissue lysates, ensuring precise and targeted measurements. Adhering to these guidelines guaranteed the reliability and reproducibility of the results.

### 4.5. Functional Assessment of Released HGF: Scratch and Scatter Assays

#### 4.5.1. Experimental Implementation of the Scratch Assay

The scratch assay was performed to evaluate the migratory properties of MSC in dependency of possible present HGF in media. MSCs were initially seeded at a density of 5000 cells/cm^2^ in a 96-well plate and allowed to grow until they reached confluency. The scratching was carried out using the Incucyte^®^ WoundMaker, a specialized 96-pin mechanical device from Sartorius (Göttingen, Germany). This device is designed to create uniform scratch wounds approximately 700–800 microns wide in cell monolayers on Incucyte^®^ ImageLock 96-well microplates. The WoundMaker is noted for its ease of use and ability to create wounds quickly without harming the cells or damaging the plastic or biomatrix of the wells.

Following the creation of the scratch wounds, the cells were treated with conditioned media derived from various fibrinogen compositions that had been incubated for 24 h to ensure the possible release of HGF into the media. The healing process was then monitored through live cell imaging over a period of 36 h. The analysis focused on the percentage closure of the scratch, providing insights into the healing capacity influenced by the different conditioned media. MSC were isolated and cultured following established protocols [43].

#### 4.5.2. Experimental Implementation of the Scatter Assay

The scatter assay was conducted using the MDCK cell line, which is frequently utilized in our laboratory due to its robustness and reproducibility in various assays. For this specific test, MDCK cells were seeded at a density of 5500 cells/cm^2^ in 48-well plates and allowed to adhere for 24 h to ensure stable cell attachment and initial colony formation.

Following the initial adhesion period, conditioned media from different fibrin gel compositions containing HGF, which had been in contact with the media for 24 h, were added to the wells. This procedure aimed to assess the influence of HGF-enriched media on the scattering behavior of MDCK cells, which is indicative of cell motility and migration.

Cell scattering was monitored for 24 h using a CellCyte^®^ live cell imaging platform (Cytena, Freiburg im Breisgau, Germany). The primary analysis involved measuring the surface area covered by individual MDCK colonies. The measurements were repeated three times, to obtain reliable results. To ensure comparability between different experimental conditions, these values were normalized. This normalization is crucial because not every colony of cells is the same size at the initial time point (timepoint 0), and differences in initial colony size could influence the perception of growth and scattering behavior. This approach allows for an accurate representation of cell scatter influenced by each HGF-containing fibrin gel.

### 4.6. Immunofluorescence Staining and Microscopy

For the immunofluorescence analysis using two-photon microscopy, a detailed staining process was followed to visualize the cytoskeletal structure and nuclei of iMSC. Initially, the phalloidin working solution was prepared by diluting 1 μL of the 1000× phalloidin conjugate stock solution into 1 mL of PBS mixed with 1% BSA. This mixture was well agitated by pipetting, creating enough staining solution for 10 chambers, with 100 μL allocated per chamber.

For staining, iMSC were grown on ibidi chamber slides for 24 and 72 h. The cell culture medium was carefully aspirated to avoid dislodging the cells, followed by a single wash with PBS. Cells were then fixed using paraformaldehyde at room temperature for 15 min. Cells were washed three times with PBS to remove residual fixative. To block non-specific binding, cells were incubated with 1% BSA for 20 min, followed by two more washes in PBS.

Next, cells were permeabilized with 0.1% Triton X-100 for 5 min, washed twice with PBS, and then treated with 100 µL of phalloidin-conjugate working solution. They were incubated at room temperature for 60 min to allow for thorough staining of the actin filaments. After this, cells were rinsed 2–3 times with PBS, with each wash lasting 5 min.

To stain the nuclei, DAPI was added, and cells were incubated for 5 min in the dark. Following this incubation, the staining solution was removed, and cells were washed three times in PBS to clear any excess stain. The preparation concluded with the cells being either immediately observed at an excitation/emission of 493/517 nm or kept covered with PBS until observation. This method ensured detailed visualization of cellular structures for further analysis.

#### Scanning Electron Microscopy

For SEM analysis, samples were fixed in glutaraldehyde, rinsed in PBS, dehydrated in a graded ethanol series, critical point dried, mounted on SEM stubs, gold sputtered and imaged using scanning electron microscopy.

### 4.7. Quantification and Statistical Analysis

For the quantification and statistical analysis of the experimental results, several specialized software tools were utilized. Graphs and visual data representations were created using GraphPad Prism (Version 10.2.2), a powerful tool for biostatistical analysis and graphing. To ensure reliable results, we always made a triplet approach (*n* = 3) and conducted statistical analysis using GraphPad. Specifically, *t*-tests or ANOVA were performed to determine the significance of differences, with *p*-values calculated accordingly.

The scratch assay analysis was facilitated by the Wound Healing Size Tool [44], a plugin for ImageJ/Fiji^®^ (Version 1.53e). This plugin specifically aids in accurately measuring the size of wound healing in scratch assays, allowing for precise quantification of cell migration over time. In the scatter assay, ImageJ was employed to determine the surface area of each cell colony. This analysis provided the necessary data to perform a *t*-test, which was used to analyze significant differences between the experimental conditions, ensuring that any observed effects were statistically validated. For the results obtained from two-photon microscopy, the post-processing of images was carried out using the Bioformat Importer plugin for ImageJ. This plugin is essential for handling and analyzing the complex image data produced by two-photon microscopy, facilitating detailed examination of cellular structures. Together, these tools provided a comprehensive approach to analyzing and presenting the data from the various assays, ensuring that the conclusions drawn were both accurate and statistically robust.

## Figures and Tables

**Table 1:** Composition of fibrin-based hydrogels. Quantities given for 48-well plates. For gels with HGF, the HGF was dissolved in GBSH5 to maintain the overall volume.

	Gel without HGF	Gel with 75 ng/mL HGF	Gel with 200 ng/mL HGF
Component	µL per Well		
Fibrinogen (10/20/40 mg/mL)	156	156	156
CaCl_2_ (50 mM)	10	10	10
GBSH_5_ incomplete	24	8.25	3
HGF in GBSH_5_	0	15.75 Stock: 1000 ng/mL	21 Stock: 2000 ng/mL
Thrombin (10 U)	20	20	20

## Data Availability

The data presented in this study are available on request from the corresponding author (accurately indicate status).
